# Phenotypic and Genetic Characterization for Incompatible Cross-Match Cases in the Feline AB Blood Group System

**DOI:** 10.3389/fvets.2021.720445

**Published:** 2021-09-13

**Authors:** Yumiko Uno, Masami Yaguchi, Tasuku Kobayashi, Eri Onozawa, Kazuhiko Ochiai, Karin Yoshida, Chihiro Nakamura, Chihiro Udagawa, Toshinori Omi

**Affiliations:** ^1^Faculty of Veterinary Science, Nippon Veterinary and Life Science University, Tokyo, Japan; ^2^Anifa Animal Hospital Kashiwa, Chiba, Japan; ^3^Research Center for Animal Life Science, Nippon Veterinary and Life Science University, Tokyo, Japan; ^4^Veterinary Medical Teaching Hospital, Nippon Veterinary and Life Science University, Tokyo, Japan; ^5^Japan Small Animal Medical Center, Saitama, Japan

**Keywords:** cat, AB blood group, *CMAH*, cross-match test, transfusion

## Abstract

The feline AB blood group system (blood types A, B, and AB) encoding the cytidine monophosphate-N-acetylneuraminic acid hydroxylase (*CMAH*) gene is the most significant in transfusion medicine and hemolysis of the newborn for cats. Blood typing and cross-matching in pre-transfusion testing are crucial to determining blood compatibility and thus prevent hemolytic transfusion reactions. We here performed serological and genetic investigations to characterize blood samples from cats with discordant results for card agglutination (CARD) and the alloantibody agglutination test for blood typing in two cats (subjects K and R). Subject K showed incompatible cross-matching in pre-transfusion testing. Red blood cells from subjects K and R determined blood type B from the CARD method showed blood type AB by alloanti-A and alloanti-B antibodies in agglutination testing. Genomic DNA sequencing of the coding region (exons 1a to 14) for the cat *CMAH* gene showed that subject K had four mutations with heterozygosity at c.139C>T, c.179G>T, c.327A>C, and c.364C>T. Similarly, the *CMAH* gene of subject R carried six mutations with heterozygosity at c.142G>A, c.187A>G, c.268T>A, c.327A>C, c.773G>A and c.1603G>A, representing a new diplotype including a novel synonymous single nucleotide polymorphism (SNP) in exon 7 (c.773 G>A: Arg258Gln). The *CMAH* diplotype in subjects K and R was different from major diplotype in blood type B cats. This study is the first to report *CMAH* variants in cats with discordant blood types between CARD and TUBE methods. These results could assist in the classification of feline AB blood types for transfusion medicine to avoid blood incompatibilities.

## Introduction

AB blood group antigens are the most significant in transfusion medicine and neonatal isoerythrolysis (NI) for cats (1–5). The feline AB blood group system is based on A and B antigens and contains type A, type B, and the rare type AB. Type A erythrocytes express N-glycolylneuraminic acid (Neu5Gc) and type B erythrocytes express N-acetylneuraminic acid (Neu5Ac) (6, 7). Cat serum contains naturally occurring antibodies against other erythrocyte antigens, with 95% of type A cats showing antibodies to type B antigen, and 35% of type B cats showing antibodies to type A antigen (2). Type AB erythrocytes express both Neu5Gc and Neu5Ac, and cats of this type show no naturally occurring antibodies to blood types A or B (2).

The enzyme cytidine monophosphate-N-acetylneuraminic acid hydroxylase is encoded by the *CMAH* gene, which changes Neu5Ac to Neu5Gc (8, 9). Humans only have Neu5Ac, because a 92-bp in exon 6 of the *CMAH* gene renders the resulting protein inactive (10, 11). Conversely, many DNA polymorphisms have been described in the cat *CMAH* gene, including c.139C>T, c.142G>A, c.179G>T, c.187A>G, c.268T>A, c.327A>C, c.364C>T, c.374C>T, c.376G>A, c.593A>C, c.868A>C, c.898A>G, c.933delA, c.1322delT, c.1342G>A, and c.1603G>A in the coding region of the cat *CMAH* gene (12–16). In our previous report, we regard that double haploids selected from multiple recessive alleles carrying one or several of these SNPs are presumed to lead to a loss or reduction of enzyme activity for synthesizing Neu5Gc from Neu5Ac, and would thus be associated with expression of Neu5Ac on the erythrocyte membrane in type B and AB cats (15).

Blood typing and cross-matching based on pre-transfusion testing are crucial to determining blood compatibility and thus preventing hemolytic transfusion reactions. There are some discrepancies in results of blood typing among different blood-typing kits depending on the sensitivity and specificity for detection of antigen (17–21). In addition, the FeLV-related anemia, other anemic cases, autoagglutination or mixed-field agglutination could affect the results of blood typing (17, 19, 20). In the present study, we describe the detection of an incompatible cross-match case (subject K) in the feline AB blood group system, and another cat (subject R) with discordant results from the card agglutination (CARD) method and alloantibody agglutination test for blood typing. We here describe our results from serological and genetic investigations to characterize blood samples from these two cats. We then characterized the low-frequency haplotypes carried according to six mutations and a novel haplotype with a new SNP in the feline *CMAH* genes of these cats.

## Methods

### Animals

#### Subject K

Subject K was a 4-year-old, male, neutered, domestic Scottish Fold cat brought in as a volunteer feline blood donor with blood type B (CARD method) according to the feline AB blood group system. Subject K showed incompatible major cross-match and compatible minor cross-match with a candidate to receive transfusion of type B blood (CARD method) in pre-transfusion testing at Kariya Animal Hospital (Tokyo, Japan).

#### Subject R

Subject R was a male, neutered, and hybrid about 1 year old identified as a B-type (CARD method) cat in a blood group screening test at our laboratory. Blood had been collected at Shippo Animal Hospital (Yokohama, Japan) with the consent of the owner.

### Serological Analyses

For this study, subjects K and R were identified due to a re-analysis of blood typing. Blood types were determined using RapidVet-H Feline Blood Type Cards according to the protocol described by the manufacturer (Kyoritsu Seiyaku Corporation, Tokyo, Japan). In addition, antigens and natural antibodies were tested by the tube (TUBE) agglutination method. Blood group antigens were also determined in subjects K and R using anti-A and anti-B alloantibodies in plasma from type B cats and type A cats, respectively. Natural antibodies in subjects K and R were tested using red blood cells (RBCs) from type A and B cats. RBC antigens from subject K were only characterized using the absorption test because of small amount of blood from subject R. Plasma containing anti-A alloantibodies and 3% RBCs from type A cats were mixed and reacted in 12 × 75-mm tubes at room temperature, and centrifuged at 3,000 rpm for 15 s. Tube agglutination testing was then performed using plasma supernatant that excluded anti-A alloantibody and a solution of 3% RBCs from subject K. Agglutination was considered positive if RBCs remained agglutinated after tubes were gently shaken.

### Molecular Analysis

Genomic DNA was extracted from whole blood using the Puregene kit (Qiagen, Valencia, CA, USA), according to the instructions from the manufacturer. The coding region of the cat *CMAH* gene was amplified from genomic DNA as described in our previous report (15). Sequencing was performed directly on PCR products, purified using the High Pure PCR Product Purification Kit (Roche, Mannheim, Germany). Using the BigDye Terminator kit (v3.1; Applied Biosystems, Foster City, CA, USA), sequencing was performed on an ABI 3730 Genetic Analyzer (Applied Biosystems). BigDye Xterminator Purification kits (Applied Biosystems) were used to purify dye-labeled fragments in accordance with the instructions from the manufacturer.

## Results

### Serological Analysis

The results of serological analysis in subjects K and R are in Table 1. Subject K showed incompatible major cross-match and compatible minor cross-match with a candidate for transfusion with blood type B in pre-transfusion testing. The major cross-match between RBCs from subject K and plasma from 10 blood-type B cats were tested using the TUBE method. Subject K showed incompatible major cross-match for all blood-type B cats. Then, the blood type of subject K was investigated by the CARD method using RapidVet-H Feline Blood Type CARD kits (Kyoritsu Seiyaku Corporation) and the TUBE method using plasma anti-A and anti-B alloantibodies from cats with type B blood and type A blood, respectively. The RBCs from subject K with blood type B as determined by the CARD method showed agglutination not only with anti-A alloantibody, but also with anti-B alloantibody by the TUBE method. These results suggested that subject K was identified as a type B cat by the CARD method but was actually a type AB cat. A less sensitive detection of A antigen using the CARD method has been reported in previous papers (17, 19, 20). Whether A antigen was expressed on RBC membranes in subject K was confirmed by the absorption test. Agglutination of A antigen on the RBC membrane on exposure to plasma from type B cats containing anti-A antibody disappeared when the anti-A antibody in plasma from type B cats was absorbed by reaction with type A RBCs.

**Table 1:** Serological investigation of subjects K and R with blood type B as determined by the CARD method.

		**CARD method**		**TUBE method** **Plasma from types ** **A and B**		**TUBE method** **RBCs from types ** **A and B**		**Plasma (absorption test)** ^*^	
								**Before**	**After**
		**Anti-A antibody (MoAb)**	**Anti-B antibody (lectin)**	**Anti-A antibody**	**Anti-B antibody**	**A antigens**	**B antigens**	**Anti-A antibody**	**Removed Anti-A antibody**
Subject K	RBCs	Negative	Positive	Positive (10)	Positive (1)	-	-	Positive	Negative
	Plasma	-	-	-	-	Negative (6)	Negative (2)	-	-
Subject R	RBCs	Negative	Positive	Positive (1)	Positive (1)	-	-	-	-
	Plasma	-	-	-	-	Negative (1)	Negative (1)	-	-
Blood type	Blood type B (only B antigen)			Blood type AB (both A and B antigens)				

*( ): number of cats used plasma or RBCs.*

* *TUBE method*.

Subject R was identified as a type B cat in laboratory blood group screening with SNP genotyping in the feline *CMAH* gene. Similar to subject K, subject R had been identified by the CARD method as a type B cat, but as a type AB cat by the TUBE method using alloantibodies. Neither subject K nor subject R carried both anti-A and anti-B alloantibodies in plasma.

### *CMAH* Gene Analysis

To characterize the *CMAH* gene, which is associated with feline AB blood group, we performed genomic DNA sequencing of the coding region (exons 1a to 14) in subjects K and R. Among the 17 single nucleotide variants (SNVs) reported for the feline *CMAH* gene, subject K showed four mutations with heterozygosity at c.139C>T (Arg47Cys) and c.179G>T (Val48Met) in exon 2, and c.327A>C (Glu109Asp) and c.364C>T (Pro122Ser) in exon 4 (Table 2). The *CMAH* diplotype (Dip.) of subject K was identified as Dip. 9 in our previous study (15), as identified from a cat with blood type B determined by the CARD method. That previous cat with Dip.9 was not blood typed using the TUBE method.

**Table 2:** Cat *CMAH* gene variants in cats showing inconsistencies between CARD method and alloantibody tests.

**SNP**	**Amino acid**	**Exon**	**Diplotype**		**Reference**
			**Subject K**	**Subject R**	
c.139C>T	Arg47Cys	2	CT	CC	(13, 15, 16)
c.142G>A	Val48Met	2	GG	GA	(12–16)
c.179G>T	Gly60Val	2	GT	GG	(15–17)
c.187A>G	IIe63Val	2	AA	AG	(15, 16)
c.268T>A	Try90Asn	3	TT	TA	(12, 14–17)
c.327A>C	Glu109Asp	4	AC	AC	(15, 16)
c.364C>T	Pro122Ser	4	CT	CC	(14–17)
c.374C>T	Ser125Leu	4	CC	CC	(16)
c.376G>A	Glu126Lys	4	GG	GG	(16)
c.593A>C	His198Pro	5	AA	AA	(16)
c.773G>A	Arg258Gln	7	GG	GA	This study
c.868A>C	Thr290Pro	8	AA	AA	(16)
c.898A>G	Lys300Glu	8	AA	AA	(16)
c.933delA	Ala312Hisfs*6	8	AA	AA	(16)
c.1322delT	Leu441*	11	TT	TT	(16, 17)
c.1342G>A	Val448Ile	11	GG	GG	(16)
c.1603G>A	Asp D535Asn	12	GG	GA	(12, 14–16)
Diplotype			Dip. 9 (15)	This Study	1
w					
Blood Type	CARD method		B	B	
	TUBE method (alloantibody)		AB	AB	

The *CMAH* gene of subject R carried six mutations, with heterozygosity at c.142G>A (Val48Met) and c.187A>G (IIe63Val) in exon 2, c.268T>A in exon 3, c.327A>C in exon 4, c.773G>A (Arg258Gln) in exon 7 and c.1603G>A (Asp535Asn) in exon 13 (Table 2). The SNP locus at c.773 G>A in exon 7 was a novel variant for the feline *CMAH* gene, representing a new diplotype for the *CMAH* gene.

## Discussion

Blood typing and cross-match testing are the most common procedures applied in effective and safe transfusion medicine. Normally, identification of matching blood types between donors and recipients prevents major and minor transfusion reactions in the feline AB blood group system. Classification of the blood type is thus very important. This study is the first to report *CMAH* variants in cats with discordant blood types between CARD and TUBE methods in the feline AB blood group system.

Type AB erythrocytes show decreased expression of both A and B antigens compared with type A or type B erythrocytes (6). Griot-Wenk et.al showed that the mean fluorescence of AB cells was half that seen with A or B cells alone (7). We here identified two cats (subjects K and R) that were blood type B according to the CARD method, but were blood type AB according to the TUBE method. Seth et al. also reported discordant results from several commercially available serological tests for blood typing as compared with results from the TUBE method (17–21). Among six of 58 discordant cases, three cats showed type B with the CARD method and type AB with not only the TUBE method, but also with immunochromatographic cartridge (CHROM), gel-based (GEL), and conventional slide (SLIDE) methods. These results suggested that the CARD method did not detect expression of A antigen in cases of blood type AB cat with extremely low levels of A antigen (17). Type AB erythrocytes have approximately half as much A and B antigen as A and B erythrocyte, respectively (6, 7). We consider that type AB cats with a weak A antigen (henceforth A_w_B) exist at low frequency among cats categorized as type B by the CARD method. We recommend both CARD method and TUBE method to determine the blood type for incompatible cross-match cases.

Many DNA polymorphisms in the cat *CMAH* gene have been described (12–16). Subjects K and R with type AB did not carry the critical genotype for blood type B (c.268:AA) and type AB (c.364:TT) cats in the *CMAH* gene (12, 14–16). The diplotype of subject K corresponded to Dip.9, which we identified in a type B cat in a previous report (15). That previous cat with Dip.9 was not blood typed using the TUBE method, so the possibility remains that Dip.9 cats are uniformly A_w_B cats. The Dip.9 type B cat in that previous study (15) was originally planned to be bred as a donor cat for type B cats in our Veterinary Medical Teaching Hospital. However, the cat was determined to be unsuitable as a donor cat and was transferred to a foster family. The present subject R with new SNPs and a new diplotype not only showed that further *CMAH* variants exist in cat populations, but also that type A_w_B cats can show genetic variation. This study suggested that not only blood typing methods and cross-matching methods but also *CMAH* variant analysis are useful in identifying appropriate blood sources for AB blood transfusion medicine.

## Data Availability Statement

The original contributions presented in the study are included in the article/supplementary files, further inquiries can be directed to the corresponding author.

## Ethics Statement

The animal study was reviewed and approved by the Experimental Animal Ethics Committee at NVLU. Written informed consent was obtained from the owners for the participation of their animals in this study.

## Author Contributions

YU, MY, TK, KY, and TO: conceived and designed the experiments. YU, MY, TK, EO, KO, CN, and TO: performed the experiments. TO and MY: analyzed the data. YU, MY, CU, and TO: wrote the paper. All authors read and approved the final manuscript.

## Funding

This work was supported by KAKENHI scientific research grants from the Ministry of Education, Culture, Sports, Science and Technology of Japan (Nos. 16K08062 and 19K06392).

## Conflict of Interest

The authors declare that the research was conducted in the absence of any commercial or financial relationships that could be construed as a potential conflict of interest.

## Publisher's Note

All claims expressed in this article are solely those of the authors and do not necessarily represent those of their affiliated organizations, or those of the publisher, the editors and the reviewers. Any product that may be evaluated in this article, or claim that may be made by its manufacturer, is not guaranteed or endorsed by the publisher.
